# MicroRNA-139 inhibits hepatocellular carcinoma cell growth through down-regulating karyopherin alpha 2

**DOI:** 10.1186/s13046-019-1175-2

**Published:** 2019-05-02

**Authors:** Ying Zan, Baofeng Wang, Liang Liang, Yujiao Deng, Tian Tian, Zhijun Dai, Lei Dong

**Affiliations:** 1 Department of Oncology, the Second Affiliated Hospital, Xi’an Jiaotong University, Xi’an, 710004 China; 2 Department of Gastroenterology, the Second Affiliated Hospital, Xi’an Jiaotong University, Xi’an, 710004 China

**Keywords:** Survival, Epithelial-to-mesenchymal transition, Colony formation, Migration and invasion

## Abstract

**Background:**

MicroRNA-139-5p (miR-139) has been shown to play important roles in hepatocellular carcinoma (HCC) development. However, the exact mechanism of miR-139 in HCC remains largely unknown.

**Methods:**

We investigated the function in human cell lines and patient tissue samples by experimental techniques in molecular biology including Co-IP assay, cell viability assay, quantitative real-time-PCR, et al. In addition, datasets were used to verify the results by database analysis. Statistical analysis was performed by using the GraphPad Prism 6 (GraphPad Software Inc., USA). A *P* value < 0.05 was defined as statistically significant.

**Results:**

In this study, we found that miR-139 was significantly down-regulated in HCC. MiR-139 level was negatively associated with the stage of HCC, and HCC patients with higher miR-139 level had longer overall survival (OS) than these having lower miR-139 expression. Overexpression of miR-139 led to reduced cell viability, elevated apoptosis, and decreased colony forming, migratory and invasive capacities in HCC cells, while down-regulation of miR-139 led to opposite phenotypes. MiR-139 also inhibited HCC growth in a xenograft mouse model. We identified karyopherin alpha 2 (KPNA2) as a direct target of miR-139. KPNA2 is up-regulated in HCC and higher KPNA2 level is associated with poor patient prognosis. Silencing of KPNA2 expression led to similar phenotypic changes as miR-139 overexpression. Restoration of KPNA2 attenuated the suppressive effects of miR-139 overexpression on cell viability, apoptosis, colony formation, migration and invasion. In addition, miR-139 overexpression and KPNA2 depletion led to decreased nucleus level of POU class 5 homeobox 1 (POU5F1) and c-myc, two well-known pro-oncogenes.

**Conclusion:**

In together, these data revealed the essential roles of the miR-139/KPNA2 axis in HCC.

## Background

Hepatocellular carcinoma (HCC) is the most common type of primary liver malignancy, which represents the sixth most prevalent cancer and the third leading cause of cancer-related death worldwide [1–3]. In spite of advancement in HCC treatment in recent years, HCC still has a poor 5-year survival rate of ~ 7% [4]. The high mortality rate of HCC is mainly due to late diagnosis at advanced stages and tumor recurrence and metastasis after surgical resection. Understanding the molecular mechanisms underlying HCC formation and development is of crucial significance for developing novel therapeutic strategies and for improving the outcome of patients with advanced HCC.

In recent years, a large body of evidence has revealed that deregulated microRNAs (miRNAs) play important roles in cancer. MiRNAs belong to a class of endogenous non-coding small RNAs that regulate gene expression post-transcriptionally. They bind to the 3′ untranslated region (UTR) of target messenger RNAs (mRNAs) to promote mRNA degradation and/or translational repression. Through down-regulating the expression of target genes, miRNAs play pivotal roles in a number of physiological and pathological processes, such as cell differentiation, proliferation, apoptosis, migration and carcinogenesis [5]. Accumulating studies have indicated that aberrant expression of specific miRNAs is implicated in HCC development and progression [6, 7]. For example, miR-122, a liver-specific miRNA, is frequently down-regulated in HCC [8] and inhibits tumor growth by down-regulating cyclin G1 [9]. MiR-139-5p (denoted thereafter as miR-139) is another miRNA that plays important roles in HCC. It is located within the second intron of the *phosphodiesterase 2A (PDE2A)* gene on chromosome 11q13.4 [10] and is often under-expressed in HCC. MiR-139 mainly functions as a tumor suppressor in HCC; it can suppress the proliferation, migration and invasion of HCC cells and induce HCC cell apoptosis via down-regulating a number of target genes, such as *T-cell factor-4 (TCF-4)* [11], *Rho-kinase 2 (ROCK2)* [12], *zinc finger E-box binding homeobox 1(ZEB1)* and *ZEB2* [13]. Notably, the number of studies of miR-139 in HCC is still very limited and the function(s) of miR-139 in HCC development remains largely unknown. Therefore, further investigation in the role of miR-139 in HCC is of essential significance.

Karyopherin alpha 2 (KPNA2) is a member of the importin α family, which plays an important role in mediating nucleocytoplasmic transport [14]. KPNA2 recognizes the nuclear localization signal (NLS) of the cargo proteins and acts as an adaptor to deliver them to the nucleus [14]. KPNA2 has been reported to be involved in the pathogenesis of variety types of cancer. KPNA2 is upregulated in multiple kinds of malignancies and high KPNA2 level is associated with adverse outcome of patients with breast cancer [15], colorectal cancer (CRC) [16], and urothelial carcinoma [17] and so forth. The biological functions of KPNA2 have been involved in promoting cancer cell proliferation, colony formation, migration and invasion and in suppressing apoptosis [18–20]. It has been shown that KPNA2 could promote carcinogenesis mainly through the nucleus translocation of cancer-associated proteins, such as POU class 5 homeobox 1 (POU5F1) [20], c-myc [18] and TP53 [21]. Regarding HCC, the clinical significance of aberrant expression of KPNA2 is unknown. However, KPNA2 has been shown to promote HCC cell growth and accelerate cell cycle progression, suggesting an oncogenic role of KPNA2 in HCC [22, 23]. Notably, the number of studies that have investigated the role of KPNA2 in HCC is very limited. Therefore, in this study we also investigated the clinical significance and biological effects of KPNA2 in HCC. KPNA2 is predicted as a direct target of miR-139 by bioinformatic tools and several high-throughput studies also indicated that miR-139 could target KPNA2 [24–26]; therefore we investigated whether miR-139 could target KPNA2 and whether KPNA2 contributed to the cellular functions of miR-139 in HCC.

In this study, we further explored the clinical significance and biological functions of aberrant expression of miR-139 in HCC. We also investigated the expression of KPNA2 in HCC and its correlation to the clinicopathological stage and prognosis of HCC patients. The effects of silencing KPNA2 on the cancerous phenotypes of HCC were also studied. Furthermore, we for the first time identified KPNA2 as a direct target of miR-139 and revealed that miR-139 inhibit HCC growth via down-regulating KPNA2. The results of this study indicated the essential significance of miR-139/KPNA2 axis in the formation and development of HCC and suggested this pathway as therapeutic target for HCC.

## Materials and methods

### Cell culture

Normal human liver cell line, HL-7702, and HCC cell lines, HepG2, Hep3B and SMMC7721, were obtained from the Shanghai Institute of Cell Biology (Shanghai, China). Cells were cultured in Dulbecco’s modified Eagle’s medium (DMEM; Gibco, USA) supplemented with 10% fetal bovine serum (FBS; Gibco, USA). All cell lines were maintained at 37 °C in a humidified incubator containing 5% CO_2_.

### Patient tissue samples

HCC and non-cancerous adjacent tissues were obtained from 20 HCC patients who were diagnosed and received surgery at the Department of Hepatobiliary Surgery, the second affiliated hospital of Xi’an Jiaotong University from January 2012 to June 2017. None of these patients had received chemotherapy or other treatments before surgery. Informed consent was obtained from all participants. The present study was approved by the Ethics Committee of the second affiliated hospital of Xi’an Jiaotong University.

### Transfection of miR-139 mimics and inhibitors

The human miR-139 mimic oligos, miR-139 inhibitor, and non-targeting control oligos were obtained from RiboBio (Guangzhou, China). The mimics, inhibitors and control oligos were transiently transfected into Hep3B and SMMC-7721 cells at a final concentration of 50 nM by using lipofectamine 2000 (Thermo Fisher Scientific, USA) according to the manufacturer’s instructions.

### Transfection of KPNA2 siRNA

SiRNAs targeting KPNA2 or control oligo (Qiagen, USA) were transfected into Hep3B cells using Lipofectamine 2000 (Thermo Fisher Scientific, USA) according to the manufacturer’s instructions.

### Quantitative real-time-PCR

Total RNA, including miRNA, was extracted from the cells or tissues, using Trizol Reagent (Life Technologies, USA) according to the manufacturer’s protocol. To measure the levels of mRNAs 1.0 μg total RNA was reversely transcribed into cDNA using the iScript cDNA Synthesis kit (Bio-Rad, USA) according to manufacturer’s instructions. The qRT-PCR was then performed by using the iQ SYBR Green Supermix (Bio-Rad, USA) on a CFX Real-Time PCR Detection System (Bio-Rad, USA) using the following amplification protocol: 95 °C for 3 min; followed by 95 °C for 15 s and 60 °C for 30 s (40 cycles). GAPDH was used as an internal control. The primers used for qRT-PCR are as follows: GAPDH forward: 5′- CCATGGGGAAGGTGAAGGTC-3′ and reverse: 5′-TGGAATTTGCCATGGGTGGA-3′; KPNA2 forward: 5′-CGAGTCTGCACCTATTTTGGC-3′ and reverse: 5′-GGGCAGCTGGTGTATTAGCA-3′. All reactions were performed in triplicate. Cell line-based experiments were repeated for three times. Fold changes in mRNA levels were calculated by using the 2-^*ΔΔ*^CT method. To determine the miR-139 level miRNAs were converted to cDNA and added with a universal tag by using the miScript II RT kit (Qiagen, USA) following the manufacturer’s protocol. The level of miR-139 was detected by qRT-PCR by using the miScript SYBR Green PCR kit (Qiagen, USA) according to the following PCR protocol: 95 °C for 15 min, followed by 40 cycles of 94 °C for 15 s, 55 °C for 30 s and 70 °C for 30 s as indicated by the manufacturer’s instruction. The U6 snRNA was used as an internal control. The primer for miR-139 is 5′-GACAGTGCACGTGTCTCCAG-3′; the U6 primer is 5′-GGATGACACGCAAATTCGTGAAGC-3′. All reactions were performed in triplicate. Cell line-based experiments were repeated for three times. Fold changes in miR-139 level was calculated by using the 2-^*ΔΔ*^CT method.

### Cell viability assay

Cells were seeded in 96-well plates at 5000 cells/well in triplicates. Cell viability was determined with the 3-(4,5-dimethylthiazol-2-yl)-2,5-diphenyltetrazolium bromide (MTT) assay by using the MTT Cell Proliferation and Cytotoxicity Assay Kit (Beyotime, China) following the manufacturer’s instructions. Ten μl MTT solution (5 mg/ml) was added to each well and incubated the plates for 4 h at 37 °C. And then 100 μl solubilization buffer was added to each well and the plates were incubated for overnight at 37 °C. The absorbance at 570 nm was measured by using a spectrophotometer (Molecular Devices, USA). All experiments were repeated for three times.

### Apoptosis assay by flow cytometry

Apoptosis of cells was determined by using the Annexin V/Hoechst 33258 double staining flow cytometry. Briefly, cells were washed twice with 500 μl cell staining buffer and then resuspended in binding buffer at a final concentration of 1X10 [6] cells/ml. Transfer 100 μl of cell suspension to a 1.5 ml tube, to each tube 5 μl of annexin V-FITC was added, followed by incubation for 15 min in dark at room temperature. After incubation 400 μl binding buffer was added to each tube. Before loading the samples, 8 μl of Hoechst 33258 (10 mg/ml) was added to each sample. Cell apoptosis was determined by performing Annexin V-FITC/ Hoechst 33258 double staining flow cytometry with a flow cytometer (LSR II, BD Biosciences, USA). All experiments were repeated for three times. The FlowJo software (FlowJo LLC, USA) was used to analyze flow cytometry data.

### Western blotting

Whole cell lysates were harvested with RIPA lysis buffer (Beyotime, China). Protein concentration was measured by using the BCA assay (Beyotime, China). Thirty μg of whole cell lysate was loaded per lane and separated by sodium dodecyl sulfate polyacrylamide gel electrophoresis (SDS-PAGE). Proteins were then transferred to Immobilon-P PVDF membranes (Merck Millipore, USA). Membranes were blocked with 5% non-fat milk for 1 h at room temperature, and then incubated with primary antibodies for overnight at 4 °C. The primary antibodies used in this study included: E-cadherin (1:1000, Abcam, USA), Vimentin (1:1000, Boster, China), SNAIL1 (1:1000, Boster, China), KPNA2 (1:1000, Abcam, USA), c-myc (1:1000, Beyotime, China), POU5F1 (1:1000, Abcam, USA), and β-actin (1:2000, Abcam, USA). After incubation with either anti-mouse or anti-rabbit secondary antibodies for 1 h at room temperature, the membrane was washed for three times with TBST. The protein bands were visualized by using electrochemical luminescence (ECL) (Thermo Fisher Scientific, USA). The membrane was then imaged with a ChemiDoc XRS imaging system (Bio-Rad, USA) and analyzed by using the Image Lab software (Bio-Rad, USA).

### Colony formation assay

The colony formation capacity of cells was determined with the colony formation assay. Three hundred living cells was plated per well on 6 well plates in triplicate and maintained in complete culture medium for about 12 days until obvious colonies formed. Colonies were fixed with 70% ethanol for 5 min and then stained with Coomassie blue for 5 min. Wells were washed with H_2_O to remove residue Coomassie blue. A colony with more than 50 cells was defined as a colony and the number of colonies per well were counted.

### Cell migration assay

To determine the migratory capacity of cells, the transwell migration assay was conducted 48 h after miR-139 mimics or inhibitors transfection. Cells were resuspended in 500 μl serum-free medium and seeded in the upper chamber of transwell inserts (8 μm pores, Corning, USA) in 24-well plates. The lower chambers were filled with 750 μl of DMEM supplemented with 20% FBS. After 24 h at 37 °C, cells in the upper chamber were removed with cotton sticks and cells migrating through the membranes were stained with 0.2% crystal violet. Number of crystal violet-stained cells was then counted. The average number of cells that passed through the membrane from 20 representative fields (3 replicates per individual experiments) was counted under a phase contrast microscope.

### Cell invasion assay

Invasion ability of cells was determined with cell invasion assay. Cells were resuspended in 500 μl serum-free medium and seeded in the upper chamber of 24-well invasion transwell inserts (8 μm pores, BD Biosciences, USA). The lower chamber of the wells were filled with 750 μl DMEM supplemented with 20% FBS. After incubation for 24 h, the cells and matrigel on the upper chamber were removed with cotton sticks, and the cells that pass through the matrigel-coated membranes were stained with 0.2% crystal violet. The invasive ability of cells was quantified by counting crystal violet-stained cells. The average number of cells that passed through the membrane from 20 representative fields (3 replicates per individual experiments) was counted under a phase contrast microscope.

### Stable cell line construction and xenograft mouse model

Lentiviral vectors overexpressing miR-139 (pCDH-CMV-miR-139) was obtained from RiboBio (Guangzhou, China). An empty lentiviral vector was use as a control. The pCDH-CMV-miR-139 plasmid was mixed with the packaging plasmid psPAX2 and PMD2.G and the mixture was then co-transfected into HEK293T cells using Lipofectamine 2000 (Thermo Fisher Scientific, USA) according to the manufacturer’s protocol. Medium containing viral particles were harvested 72 h after transfection. Hep3B cells were infected with the miR-139 expressing or control lentivirus and then selected with puromycin to establish stable cell lines constitutively expressing miR-139. Hep3B cells overexpressing miR-139 or control cells were then injected subcutaneously into the hind flank of nude mice. Each group had four mice. Tumors were harvested on day 28 after injection and tumor weight was measured.

### Luciferase reporter assay

The 3’untranslated region (UTR) of the KPNA2 mRNA containing the miR-139 targeting site were cloned and inserted into psi-CHECK2 dual-luciferase reporter vector (Promega, USA) between the XhoI(5′) and NotI(3′) sites. The reporter plasmid with deleted miR-139 targeting site also was constructed. Hep3B cells were co-transfected with 1 μg of wildtype pKPNA2–3’UTR or mutant pKPNA2–3’UTR and 100 nM miR-139 mimic oligos or inhibitors. Cells were harvested 24 h after transfection and the activities of firefly luciferase and Renilla luciferase in the cell lysates were measured with the Dual-Luciferase Assay System (Promega, USA) according to the manufacturer’s instructions. The assay was performed in triplicates. The expression level of KPNA2 3’UTR was defined as Renilla luciferase activity normalized to firefly luciferase activity for each well.

### Co-IP assay

For Co-IP assays, the cells were lysed in precooled RIPA buffer. Anti-KPNA2 antibody or IgG control were conjugated to Protein A + G beads (Beyotime, China) according to the manufacturer’s instructions. The conjugated beads were rinsed for 3 times with RIPA buffer and then added into 3 tubes of each cell lysate. The immunoprecipitates were washed for 3 times with lysis buffer and then western blotting was carried out to measure c-myc and POU5F1 as described above.

### Analysis of liver hepatocellular carcinoma (LIHC) dataset

LIHC dataset containing 470 HCC samples was obtained from TCGA. Level 3 miRNA-Seq data generated from the miSeq Illumina platform and clinicopathological features of patients were retrieved from the TCGA Data Portal. All samples have clinical and follow-up information. The dataset contained 448 patients with miRNA-Seq data; therefore only patients with microRNA data were used in this analysis. As the data were obtained from TCGA, further approval by an ethics committee was not required. Expression of miR-139 or KPNA2 in patients at different disease stages was compared by using *one-way* analysis of variance (ANOVA). Overall survival (OS) was assessed by using the Kaplan-Meier method and curves were compared by univariate (*log-rank*) test. The correlation between miR-139 level and *KPNA2* mRNA level was analyzed with correlation analysis.

### Statistical analysis

Statistical analysis was performed by using the GraphPad Prism 6 (GraphPad Software Inc., USA). Two groups were compared with Student’s *t*-test, three or more treatments or groups were compared with *one-way* ANOVA followed by the Tukey–Kramer post hoc analysis. Cell line-based experiments were repeated for at least three times. Data represented the mean ± standard error (SEM) of all experiments or each group. A *P* value < 0.05 was defined as statistically significant.

## Results

### MiR-139 is down-regulated in HCC cells and tissues and associated with poor patient outcome

**Fig. 1:**
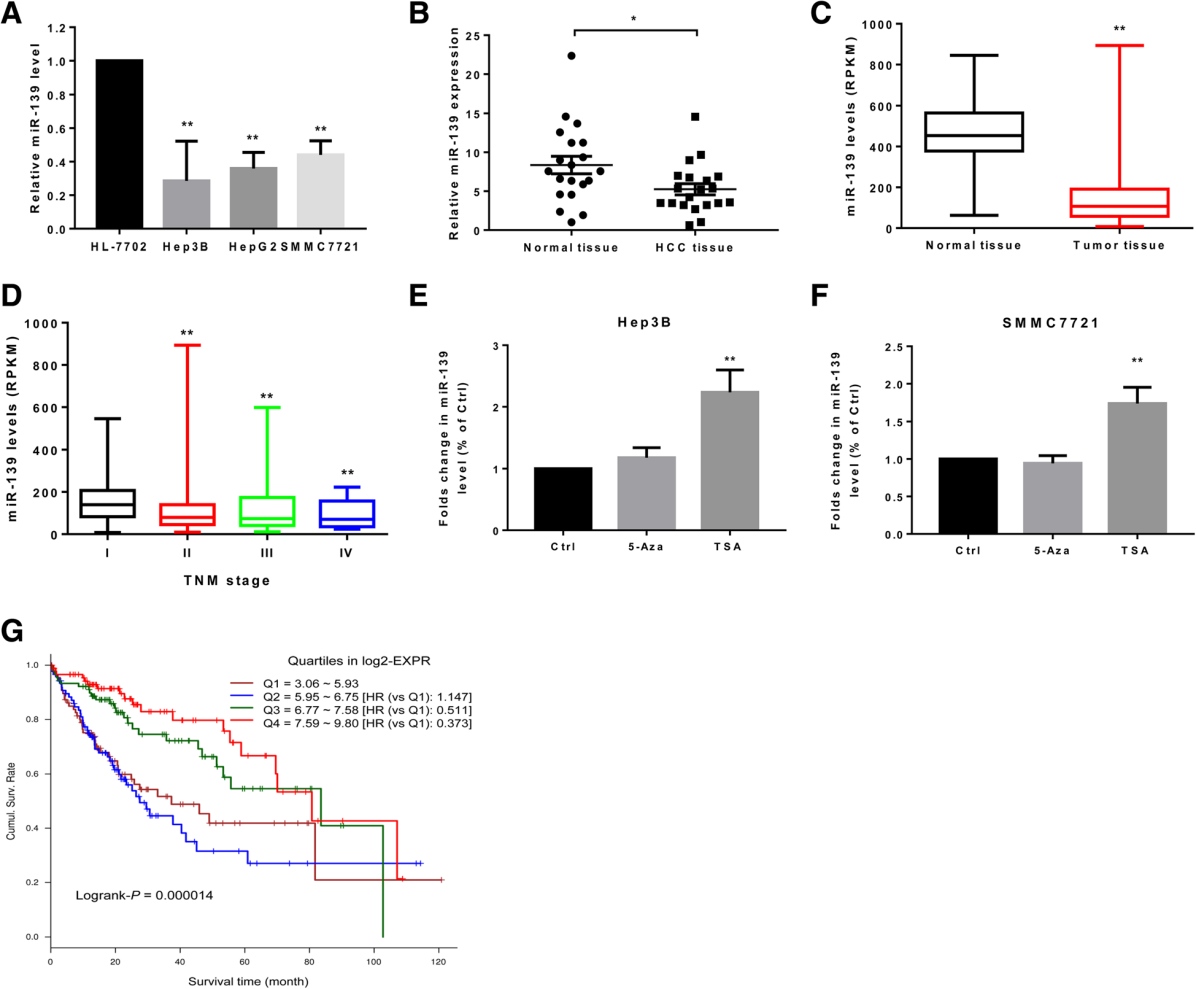
MiR-139 is down-regulated in HCC cells and tissues and associated with poor patient outcome. **a** MiR-139 level was measured in HL-7702, HepG2, Hep3B and SMMC7721 cells by qRT-PCR. **b** MiR-139 level was measured in the cancerous tissues and adjacent non-cancerous tissues in 20 HCC patients. **c** and (**d**) MiR-139 level in the LIHC dataset in TCGA was retrieved and analyzed for association with the clinicopathological features of HCC patients. **c** Mir-139 level in HCC tissues compared to non-cancerous tissues. **d** MiR-139 level in HCC patients at different pathological stages. **e** and (**f**) Hep3B and SMMC7721 were treated with DMSO, 5-Aza (5 μmol/L) or TSA (200 μmol/L) for 24 h. qRT-PCR was performed to quantify miR-139 expression. **g** The OS of HCC patients with low or high miR-139 level was analyzed with Kaplan-Meier survival analysis, and the curves were compared with the Log-rank test. The Q1–4 represent the 25% quantile of miR-139 expression. Q1 represents the lowest 25%, Q2 represents 25–50%, Q3 is the 50–75% and Q4 is the 75–100%. All experiments were repeated 3 times. * *P* < 0.05, ** *P* < 0.01

Initially, we compared miR-139 level in HL-7702 cells, a normal liver cell line, and three HCC cell lines, including HepG2, Hep3B and SMMC7721, and found that miR-139 was significantly down-regulated in HCC cell lines (Fig. 1a). We then compared miR-139 expression in the cancerous tissues and adjacent non-cancerous tissues in 20 HCC patients. The results showed that miR-139 was under-expressed in HCC tissues compared to surrounding normal tissues (Fig. 1b). In consistence, the LIHC dataset in TCGA that has a larger cohort of patients also indicated that mir-139 expression was down-regulated in HCC tissues compared to non-cancerous tissues (Fig. 1c). We also analyzed miR-139 level in HCC patients at different pathological stages. The results revealed that miR-139 level was gradually down-regulated as the stage of disease was increased (Fig. 1d), suggesting that lowered miR-139 level might be involved in the progression of HCC. To investigate the mechanism accounting for the lowered expression of miR-139, we treated Hep3B and SMMC7721 with 5-AZA, a DNA methyltransferase inhibitor or TSA, a histone deacetylase inhibitor. The results indicated that inhibition of histone deacetylase activity led to significant up-regulation of miR-139 level, but 5-AZA did not result in significant change in miR-139 level, suggesting that miR-139 is under-expressed in HCC due to lowered histone acetylation (Fig. 1e and f). Furthermore, the survival analysis discovered that HCC patients with higher miR-139 level had significantly longer overall survival (OS) than these having lower miR-139 expression (Log-rank *P* < 0.001, Fig. 1g).

### MiR-139 inhibits HCC cell growth and induces apoptosis

**Fig. 2:**
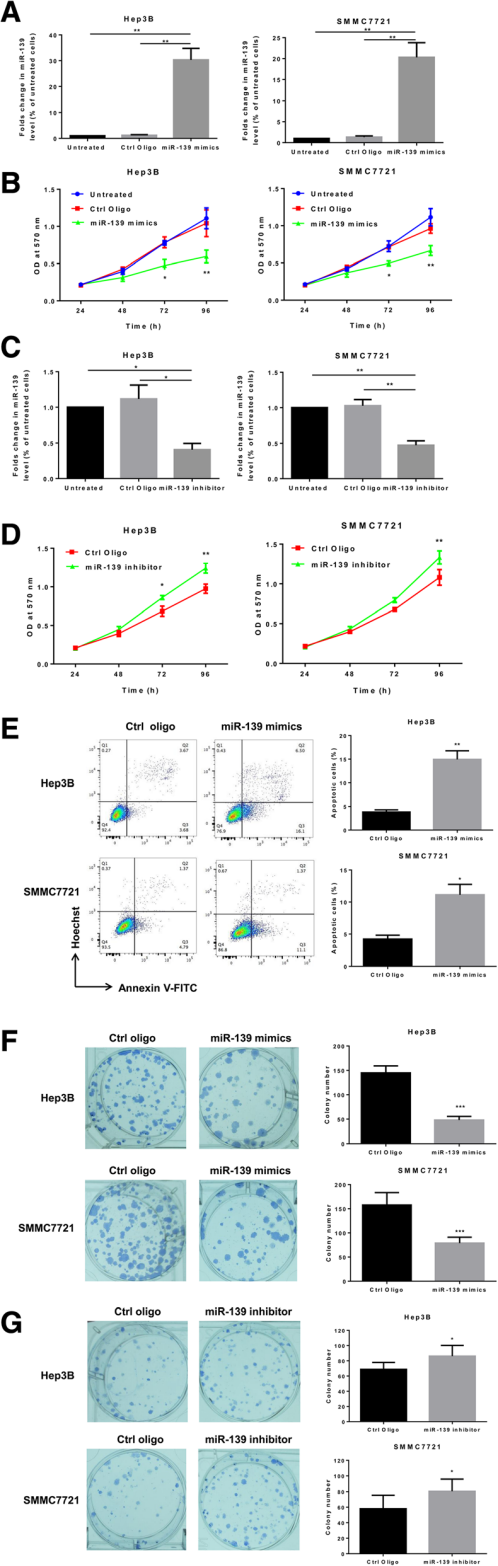
MiR-139 inhibits HCC cell growth and induces apoptosis. **a** and (**b**), Hep3B and SMMC7721 cells were transfected with 100 nM miR-139 mimic oligos or control oligos. **a** MiR-139 level was measured with qRT-PCR. **b** Cell viability was measured using MTT assay on indicated days. **c** and (**d**) Hep3B and SMMC7721 cells were transfected with 100 nM miR-139 inhibitors or control oligos. **c** MiR-139 level was measured with qRT-PCR. **d** Cell viability was measured using MTT assay on indicated days. **e** Cell apoptosis was measured with the Annxin V-FITC/Hoechst 33258 double staining flow cytometry in control cells or cells overexpressing miR-139. The cells were incubated for 72 h after transfection of the oligos. **f** Colonogenic assay was performed to measure the colony formation capacity of cells. Three hundred living cells were plated in 6 well plates in triplicate. Cells were cultured in complete medium until visible colonies were formed. Colonies were fixed with ethanol and stained with crystal violet, and the number of colonies per well were counted under a dissecting microscope. **g** Colonogenic assay was performed to measure the colony formation capacity of cells. In 6 well plates 150 living cells were plated per well in triplicate. All experiments were repeated 3 times. * *P* < 0.05, ** *P* < 0.01, *** *P* < 0.001

We then investigated the biological effects of miR-139 on the cancerous phenotypes of HCC cell lines. MiR-139 was overexpressed in Hep3B and SMMC7721 cell lines by transient transfection of miR-139 mimic oligos, and the overexpression of miR-139 was validated by qRT-PCR (Fig. 2a). The MTT assay indicated that ectopic expression of miR-139 led to significantly reduced cell viability in both cell lines (Fig. 2b). We also inhibited miR-139 expression via transient transfection of anti-miR-139 oligos (Fig. 2c). And down-regulation of miR-139 led to slightly increased viability in Hep3B and SMMC7721 cells (Fig. 2d). Furthermore, up-regulated miR-139 resulted in a substantial increase in cell apoptosis (Fig. 2e). The colony formation assay indicated that Hep3B and SMMC7721 cells overexpressing miR-139 had decreased colony formation capacity than the control cells (Fig. 2f). On the contrary, down-regulation of miR-139 promoted the colony formation capacity in these two cell lines (Fig. 2g). In together, these results suggested that miR-139 played a tumor suppressive role in HCC through inhibiting cell proliferation and inducing cell apoptosis.

### MiR-139 suppresses HCC migration and invasion

**Fig. 3:**
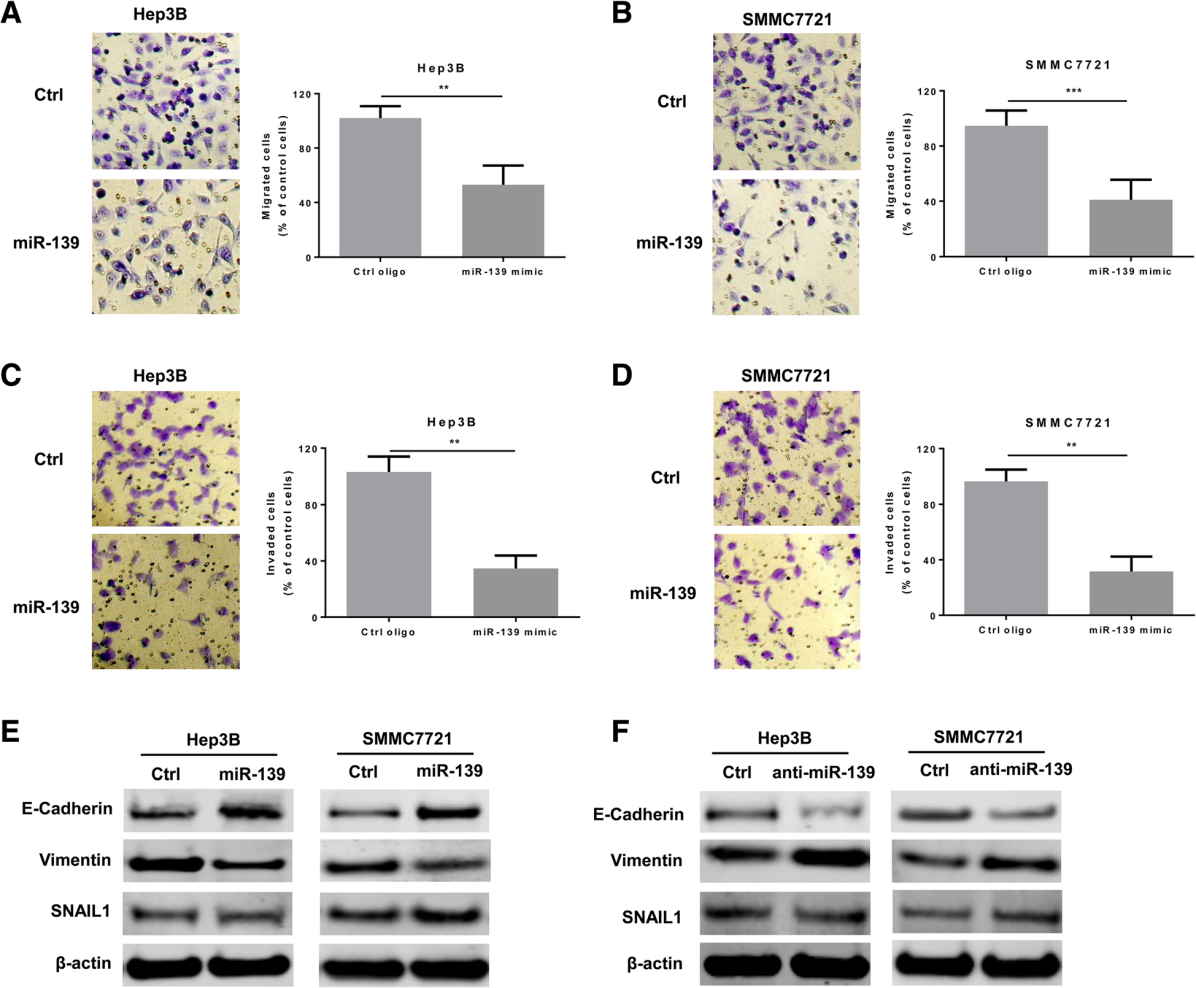
MiR-139 suppresses HCC migration and invasion. **a**-**e** Hep3B and SMMC7721 cells were transfected with 100 nM miR-139 mimic oligos or control oligos. The migration capacity of (**a**) Hep3B and (**b**) SMMC7721 cells were analyzed with the transwell migration assay. The invasion ability of (**c**) Hep3B and (**d**) SMMC7721 cells were measured with the matrigel transwell invasion assay. **e** The protein expression levels of E-cadherin, Vimentin and SNAIL1 were assayed with western blot in both Hep3B and SMMC7721 cells. **f** Hep3B and SMMC7721 cells were transfected with 100 nM miR-139 inhibitory or control oligos. The protein levels of E-cadherin, Vimentin and SNAIL1 were measured by western blot in both cell lines. All experiments were repeated 3 times. ** *P* < 0.01, *** *P* < 0.001

We also investigated the effects of miR-139 overexpression on the migration and invasion abilities of Hep3B and SMMC7721 cells. The transwell migration assay indicated that overexpression of miR-139 significantly suppressed migration of both cell lines (Fig. 3a and b). Furthermore, the invasion assay showed that ectopic expression of miR-139 also resulted in significantly attenuated invasion ability of Hep3B and SMMC7721 cells (Fig. 3c and d). Epithelial-to-mesenchymal transition (EMT) has been shown to play important roles in regulating cell migration and invasion. Therefore, we further investigated the influence of deregulated miR-139 expression in the expression of EMT markers. The results revealed that in cells overexpressing miR-139, the protein level of E-cadherin, an epithelial marker, was up-regulated, while expression of Vimentin, a mesenchymal marker, was suppressed (Fig. 3e). In addition, the level of an important inducer of EMT, snail family transcriptional repressor 1 (SNAIL1), was not affected (Fig. 3e). On the contrary, in the cells with down-regulated expression of miR-139, E-cadherin level was down-regulated, while Vimentin expression was up-regulated, and SNAIL1 expression remained unchanged (Fig. 3f). These results indicated that miR-139 might inhibit HCC cell migration and invasion via regulating the epithelial–mesenchymal plasticity.

### MiR-139 inhibits HCC growth in vivo

**Fig. 4:**
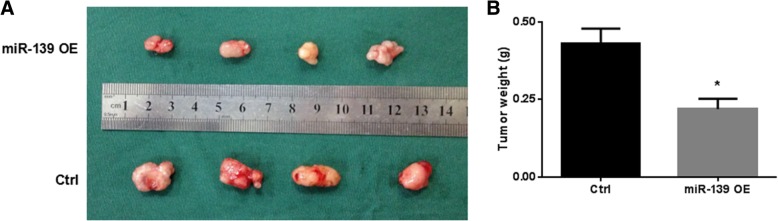
MiR-139 inhibits HCC growth in vivo*.* Hep3B stably expressing miR-139 (pLenti-miR-139) and control cells (pLenti-VC) were injected into nude mice. Each group had four mice. Tumors were harvested on day 28 after injection and tumor weight was measured. **a** A display of tumors of both groups. **b** The weight of tumors. * *P* < 0.05

To further confirm the tumor suppressive role of miR-139 in HCC, we assessed the effect of enforced expression of miR-139 on HCC cell growth in a nude mouse xenograft model. We constructed Hep3B cells stably overexpressing miR-139 with lenti-virus-mediated transduction. Hep3B cells transduced with miR-139 or control vectors were then injected subcutaneously into the hind flank of nude mice. On day 28, the tumors were harvested and weighed. The miR-139 overexpressing tumors were visibly smaller than control tumors (Fig. 4a), and their weight was significantly lower compared to that of control tumors (Fig. 4b). These results further indicated that miR-139 could inhibit HCC growth in vivo.

### KPNA2 is a direct target of miR-139

**Fig. 5:**
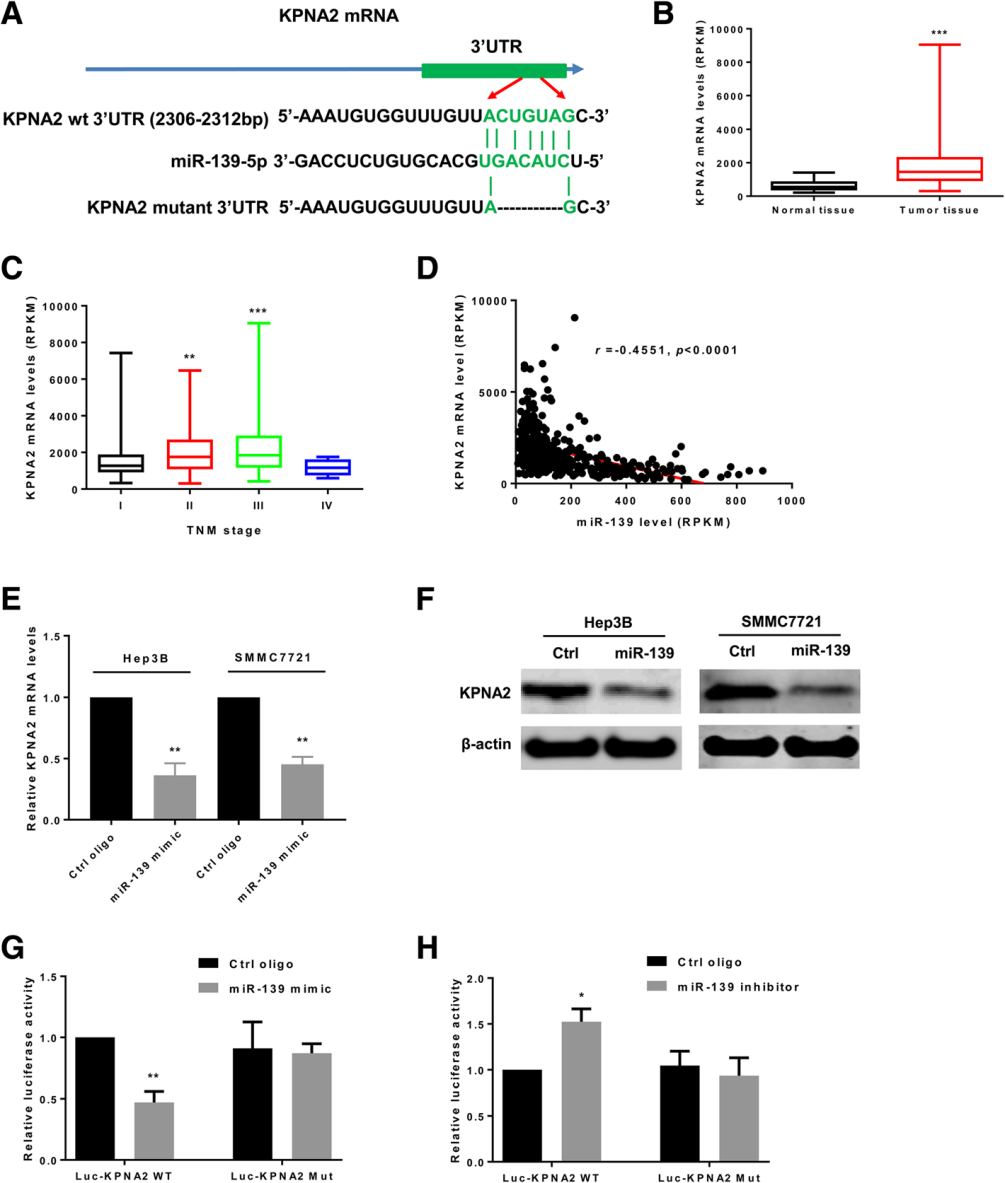
KPNA2 is a direct target of miR-139. **a** A schematic illustration of the targeting sites of miR-139 in the 3′ UTR of *KPNA2* mRNA transcript and the luciferase reporter plasmids containing parts of KPNA2 3’UTR with either a wildtype or deleted miR-139 target sites. **b**-**d** *KPNA2* mRNA level was retrieved from the LIHC dataset in TCGA. **b** KPNA2 level was compared between HCC tissues and non-cancerous tissues. **c** KPNA2 level in HCC patients at different pathological stages. **d** The correlation analysis for the association between miR-139 and KPNA2 levels. **e** and (**f**) Hep3B and SMMC7721 cells were transfected with miR-139 mimics or control oligos. The (**e**) mRNA and (**f**) proteins levels of KPNA2 were analyzed by qRT-PCR and western blot, respectively. **g** In Hep3B cells the luciferase reporter assay was performed. MiR-139 mimics and the reporter plasmids or control vectors were co-transfected into the cells. The Renilla and firefly luciferase activities of the reporters or vectors were measured with the Dual-Glo assay. **h** The anti-miR-139 oligos and the reporter plasmids or control vectors were co-transfected into the Hep3B cells. The Renilla and firefly luciferase activities of the reporters or vectors were measured with the Dual-Glo assay. All experiments were repeated 3 times. * *P* < 0.05, ** *P* < 0.01, *** *P* < 0.001

*KPNA2* mRNA transcript exhibits a putative target sequence for miR-139 in 3′ UTR and is predicted as a direct target of miR-139 by miRanda (Fig. 5a). In addition, several studies based on high-throughput sequencing also identified KPNA2 as a candidate target of miR-139 [24–26]. However, no study has established their direct regulatory relationship. Firstly, we analyzed the LIHC dataset for the correlation between miR-139 and KPNA2 level. We found that KPNA2 mRNA level in HCC tissues was significantly higher than that of non-cancerous tissues (Fig. 5b). In addition, KPNA2 mRNA level was increased in HCC patients at more advanced pathological stages (Fig. 5c). We also identified a negative correlation between miR-139 and KPNA2 levels (*r* = − 0.4551, *p* < 0.0001, Fig. 5d). We then determined the effects of miR-139 overexpression on the endogenous mRNA and proteins levels of KPNA2 in Hep3B and SMMC7721 cells. The results indicated that the mRNA (Fig. 5e) and protein (Fig. 5f) levels of KPNA2 were both significantly down-regulated in cells over-expressing miR-139 compared to control cells. To determine whether KPNA2 was a direct target of miR-139 in HCC, we constructed luciferase reporter plasmids containing parts of KPNA2 3’UTR with either a wildtype or deleted miR-139 target sites and performed the luciferase reporter assay in Hep3B cells. As shown in Fig. 5g, transfection of miR-139 mimics suppressed the Renilla luciferase activity of the reporters containing wild-type miR-139 targeting site of KPNA2, while the Renilla luciferase activity of the reporter plasmid containing deleted miR-139 target sites was not significantly reduced. On the contrary, transfection of anti-miR-139 oligos caused elevated Renilla luciferase activity of wild type luciferase reporter, but the Renilla luciferase activity of mutant reporter remained unchanged (Fig. 5h). These results confirmed that KPNA2 mRNA transcript was directly targeted by miR-139 in HCC cells.

### Down-regulating KPNA2 leads to decreased growth of HCC cells

**Fig. 6:**
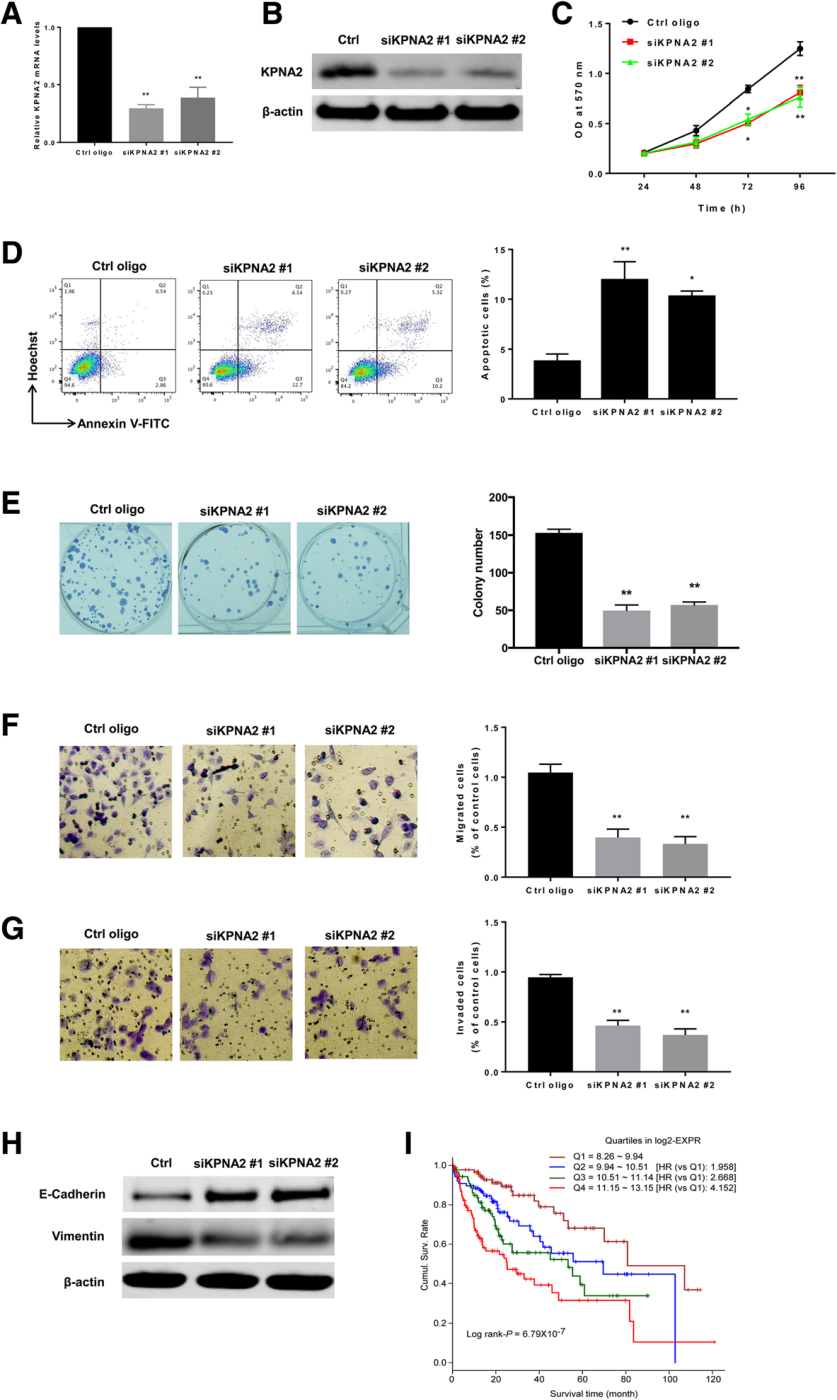
Down-regulating KPNA2 leads to decreased growth of HCC cells. Hep3B cells were transfected with non-targeting control oligo or with siRNA specifically targeting KPNA2 using lipofectamine 2000. **a** KPNA2 mRNA level was measured with qRT-PCR. **b** Western blot was used to assay the protein level of KPNA2. **c** Cell viability was measured using the MTT assay. **d** Cell apoptosis was measured with the Annxin V-FITC/Hoechst 33258 double staining flow cytometry. The cells were incubated for 72 h after transfection of the oligos. **e** colonogenic, (**f**) migratory and (**g**) invading capacities of cells were assayed as described above. **h** The protein levels of E-cadherin and Vimentin were measured using western blot. **i** The OS of HCC patients with low or high KPNA2 level was analyzed with Kaplan-Meier survival analysis and the curves were compared with the Log-rank test. The Q1–4 represent the 25% quantile of KPNA2 expression. Q1 represents the lowest 25%, Q2 represents 25–50%, Q3 is the 50–75% and Q4 is the 75–100%. All experiments were repeated 3 times. * *P* < 0.05, ** *P* < 0.01

Hep3B cells were transfected with non-targeting control oligo or with siRNA specifically targeting KPNA2. Knockdown of *KPNA2* on mRNA and protein levels was confirmed by qRT-PCR (Fig. 6a) and western blot (Fig. 6b), respectively. Cells transfected with KPNA2-specific siRNAs had significantly decreased cell viability (Fig. 6c) and increased apoptosis (Fig. 6d) than the control cells. Depletion for KPNA2 also led to decreased clonogenic (Fig. 6e), migratory (Fig. 6f) and invading (Fig. 6g) capacities in Hep3B cells compared to control cells. In addition, the protein level of E-cadherin and Vimentin was up-regulated and down-regulated, respectively, in response to depletion of KPNA2 (Fig. 6h). Regarding the significance of KPNA2 in patient outcome, HCC patients with high KPNA2 level had significantly shorter OS than patients having low KPNA2 expression (Fig. 6i).

### MiR-139 suppresses HCC growth through targeting KPNA2

**Fig. 7:**
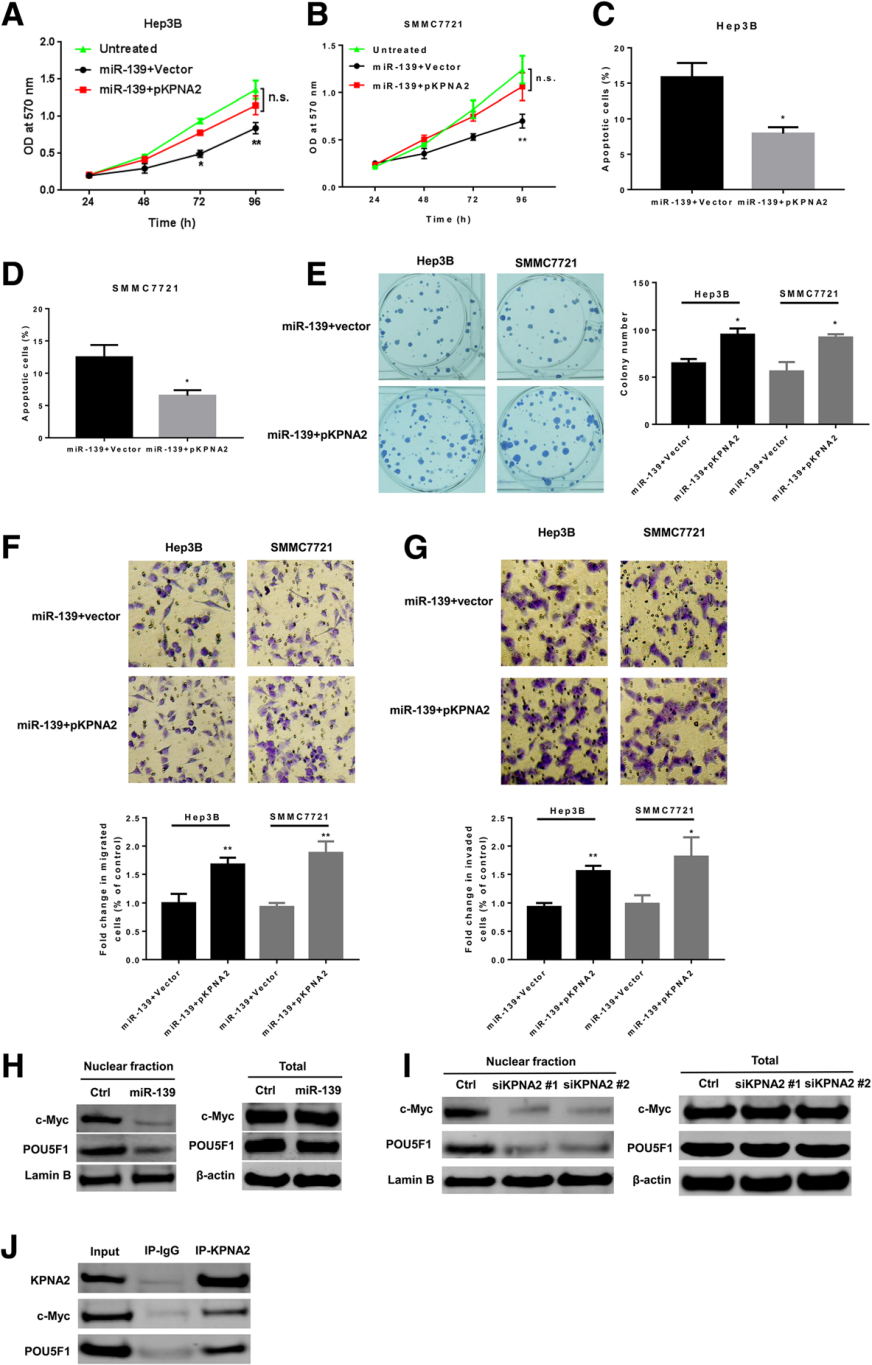
MiR-139 suppresses HCC growth partially through targeting KPNA2. Hep3B and SMMC7721 cells were transfected with miR-139 mimics and a construct encoding KPNA2 or an empty vector. Cell viability of (**a**) Hep3B and (**b**) SMMC7721 cells were measured with MTT assay at indicated time points. Apoptosis of (**c**) Hep3B and (**d**) SMMC7721 cells was measured by annexin V/Hoechst 33258 double staining flow cytometry. **c** and (**d**) SMMC7721 cells were transfected with miR-139 mimic oligos and a construct encoding DNMT1 or an empty vector. **c** Cell viability and (**d**) apoptosis were measured by MTT assay and flow cytometry, respectively. **e** Colonogenic, (**f**) migratory and (**g**) invading capacities of cells were assayed as described above. In cells transfected with (**h**) miR-139 mimics or (**i**) KNPA2 specific siRNAs, total and nucleus c-myc and POU5F1 were measured with western blot. **j** Co-IP assay detected the direct interaction between KPNA2 and c-myc and POU5F1. All experiments were repeated 3 times. * *P* < 0.05, ** *P* < 0.01

We then investigated whether miR-139 suppressed HCC growth via down-regulating KPNA2. Hep3B and SMMC7721 cells were transfected with miR-139 mimic oligos and a construct encoding KPNA2 or an empty vector. We found that restoration of KPNA2 level attenuated the inhibitory effect of miR-139 overexpression on cell viability (Fig. 7a and b) and led to decreased cell apoptosis (Fig. 7c and d). Cells co-transfected with miR-139 mimics and the KPNA2 encoding construct showed significantly increased clonogenic capacity compared to cells co-transfected with miR-139 mimics and an empty vector (Fig. 7e). In addition, restoration of KPNA2 expression significantly reversed the inhibitory effects of miR-139 overexpression on the migratory (Fig. 7f) and invasive (Fig. 7g) capacities. Since KPNA2 is involved in nucleocytoplasmic transport of proteins, we investigated the effects of miR-139 overexpression and KPNA2 depletion on the nucleus transportation of c-myc and POU5F1, which have been reported to promote tumor growth in nucleus. As shown in Fig. 7h, up-regulation of miR-139 led to decreased nucleus levels of c-myc and POU5F1, while the total protein levels of these two genes were not altered. Knockdown of KPNA2 by siRNA transfection also led to decreased nucleus levels of c-myc and POU5F1 without changing their total cellular levels (Fig. 7i). In addition, the Co-IP assay indicated that KPNA2 could directly bind to c-myc and POU5F1 (Fig. 7i). These results suggested that decreased KPNA2 level might exert function via reducing cytoplasm to nucleus transportation of c-myc and POU5F1, two well-known oncogenes that can promote tumor growth.

## Discussion

HCC is a highly malignant primary liver cancer, which has poor prognosis due to late diagnosis and unresponsiveness to therapy. In recent years, a large body of evidence has revealed that miRNAs play important roles in HCC formation and development [27]. MiR-139 has recently been reported to mainly function as a tumor suppressor in multiple types of human cancers, including HCC [28], CRC [29], and gastric cancer [30]. Although several studies have reported that miR-139 can inhibit HCC cell growth and low miR-139 level is associated with poor prognosis, its functions in HCC and the mechanism of its suppressive roles in HCC have not been fully studied. Therefore, we further investigated the role(s) of miR-139 in HCC both in vitro and in vivo in the present study.

MiR-139 expression is frequently suppressed in HCC [12, 27, 28, 31]. In this study, we also found that miR-139 was down-regulated in HCC cell lines and tissues. The reason for the down-regulation of miR-139 in cancers has been rarely studied. Epigenetic regulation has been reported to silence the expression of miR-139 at transcriptional level. Bao et al. found that CD44 binds directly to HER2 to induce deacetylation of histone H3 lysine 9 and suppresses transcription of miR-139 in gastric cancer cells [32]. Watanabe et al. has shown that miR-139 was epigenetically silenced by histone H3 lysine 27 trimethylation (H3K27me3) of its host gene PDE2A in NSCLC [33]. Furthermore, miR-139 level also can be regulated post-transcriptionally. For example, long non-coding RNA XIST [34] and LINC00152 [30] can down-regulate the expression of miR-139 via acting as “sponges” in bladder cancer and CRC, respectively. However, the mechanism for regulating miR-139 level in HCC has not been reported yet. We treated the HCC cells with 5-AZA, a DNA methyltransferase inhibitor or TSA, a histone deacetylase inhibitor. Only the TSA treatment restored the expression of miR-139 in HCC, suggesting increased histone acetylation might contribute to the down-regulation of miR-139 expression in HCC. Several studies have indicated the significance of miR-139 down-regulation in HCC. Wong et al. showed that down-regulation of miR-139 in HCC was associated with advanced tumor stages and poor prognosis [12]. The LIHC dataset in the TCGA contains a much larger cohort of HCC patients and comprehensive characterization of each sample. To further confirm the significance of under-expression of miR-139 in HCC, we analyzed the correlation between miR-139 level and clinicopathological features HCC patients based on the LIHC cohort. We also found that low miR-139 level was associated with advanced stage of the disease and dismal patient survival (Fig. 1), in consistence with the reported studies. These data strongly suggested that miR-139 plays a tumor suppressive role in HCC.

MiR-139 has been reported to be involved in a variety of cellular functions, including cell proliferation, apoptosis, cell cycle, cell migration and invasion, and through regulating these essential processes in cells miR-139 exerts its suppressive roles in tumors [35]. For example, miR-139 has been shown to suppress proliferation and induce apoptosis in CRC [30], osteosarcoma [28], gastric cancer [36], HCC [11–13], etc. In this study, we found that overexpression of miR-139 led to significantly reduced cell viability, increased apoptosis and impaired clonogenic capacity in HCC cells (Fig. 2). And down-regulation of miR-139 by transfecting inhibitory oligos caused opposite phenotypic changes (Fig. 2). In addition, miR-139 inhibited HCC growth in a nude mouse xenograft model (Fig. 4). These data are in consistence with published references, and further confirmed that miR-139 plays a tumor suppressive role in HCC.

The migratory and invasive abilities of cancer cells play essential roles in cancer metastasis, as enhanced cell migration and invasion promote distal dissemination of cancers [37]. We found that miR-139 overexpression significantly suppressed the migration and invasion abilities of Hep3B and SMMC7721 HCC cells (Fig. 3). On the contrary, inhibition of miR-139 expression resulted in enhanced migratory and invasive capacities in these two cell lines (Fig. 3). These results suggested that down-regulation of miR-139 in HCC might promote the metastasis of HCC. In consistence with our results, Qiu et al. has reported that miR-139-5p inhibits migration and invasion of HCC cells [13]. Hua et al. also showed that miR-139-5p inhibits proliferation, migration and invasion in HCC [38]. However, we did not find significant difference in miR-139 level between HCC patients with and without metastasis (data not shown). Therefore, further experimental studies are guaranteed to reveal the roles miR-139 in HCC metastasis. Epithelial–to-mesenchymal transition (EMT) has been shown to play essentials in promoting migration and invasion of cancer cells [39]. This process can be induced by several transcriptional factors, such as Snail1, zinc finger E-box binding homeobox 1 (ZEB1) and ZEB2, and twist family bHLH transcription factor 1(TWIST1) [39]. Qiu et al. indicated that miR-139 inhibited EMT in HCC by direct targeting to the 3′ UTR of ZEB1 and ZEB2, which in turn suppressed the migration and invasion of HCC cells [13]. We also measured the changes in EMT and epithelial markers in HCC cells in response to alterations in miR-139 level. The results revealed that in cells overexpressing miR-139, the protein level of E-cadherin, an epithelial marker, was up-regulated, while expression of ZEB1 and Vimentin, two EMT marker, was suppressed, while another important inducer of EMT, SNAIL1, was not affected (Fig. 3), further indicating that miR-139 can regulate migration and invasion in HCC cells via regulating EMT. However, we did not explore the mechanism underlying this effect. We will include it in our future studies. In addition to EMT, other mechanisms also have been reported to be involved in the regulation of migration and invasion by miR-139. For example, the Wnt signaling, MAPK signaling, TGFB signaling, PI3K signaling, and Notch signaling pathways were all involved in the regulation by miR-139 [35]. Yu et al. has shown that miR-139 inhibits the proliferation, migration and invasion of gastric cancer cells by directly targeting ρ-associated protein kinase 1 (ROCK1) [36]. Zhong et al. reported that miR-139 inhibits the proliferation and migration of osteosarcoma cells via targeting forkhead-box P2 (FOXP2) [28]. Notably, studies in the mechanisms for miR-139 regulation of cell migration and invasion in HCC are still very limited and these mechanisms have not been validated in HCC. Further studies are therefore required to fully understand the molecular mechanisms accounting for the miR-139 regulation of cell migration and invasion in HCC.

KPNA2 is a member of the importin α family and is thought to be an important player of nucleocytoplasmic transport [14, 40]. KPNA2 is upregulated in multiple types of cancer and associated with an adverse outcome of patients in breast cancer [15], CRC [16], urothelial cancer [17], etc. In HCC, the change in KPNA2 expression is not clear yet. We analyzed the LIHC dataset in TCGA and found that it up-regulated in HCC tissues and its level was further increased at more advanced stage. The survival analysis indicated that high KPNA2 was associated with dismal patient outcome. These results suggested a promoting role of KPNA2 in HCC progression. To investigate the biological roles of KPNA2 in HCC, we silenced KPNA2 in Hep3B cells via transfection of KPNA2 specific siRNA. Depletion for KPNA2 resulted in decreased cell viability, increased apoptosis, and compromised clonogenic ability, suggesting that KPNA2 played an oncogenic role in HCC development. These results are consistent with other studies in the role of KPNA2 in cancers. Yang et al. has reported that silencing of KPNA2 inhibited HCC cell growth and survival [23]. In other types of cancer, for example, knockdown of KPNA2 inhibited the proliferation of cells of ovarian cancer [18]. In addition, overexpression of KPNA2 in benign breast cells could increase cell colony formation ability and migration activity [41]. Furthermore, silencing KPNA2 also compromised the migratory and invading capacities of HCC cells, suggesting it was involved in HCC metastasis. E-cadherin was up-regulated and Vimentin was down-regulated in response to KPNA2 knockdown, suggesting that KPNA2 might promote HCC migration and invasion via induction of EMT. However, the mechanism for this regulation needs to be explored in future studies.

In the 3′ UTR of KPNA2 mRNA transcript, a miR-139 targeting site was predicted by miRanda, and several high throughput studies indicated that KPNA2 is a target of miR-139 [24–26]. In HCC the expression of KPNA2 is reversely correlated to the level of miR-139 and the luciferase assay further confirmed that KPNA2 is directly regulated by miR-139. As shown above, overexpression of miR-139 and depletion for KPNA2 both resulted in similar phenotypic changes in HCC cells. Taken together, these results led us to hypothesize that miR-139 exert its function via regulating the expression of KPNA2. To test this hypothesis, we restored the expression of KPNA2 by co-transfection of a plasmid that contains the encoding sequence of KPNA2 in cells overexpressing miR-139. Restoration of KPNA2 expression significantly reversed the inhibitory effects of miR-139 overexpression on cell viability, colony formation, migration and invasion, suggesting that miR-139 functions through lowering the expression of KPNA2. KPNA2 belongs to the importin α family, which plays important roles in nucleocytoplasmic transport; and KPNA2 has been reported to promote carcinogenesis through translocation of cancer-associated cargo proteins, including tumor suppressor and oncogenes. Therefore we assumed that KPNA2 might affect HCC cell via regulating nucleus transportation of other proteins. POU5F1, also known as OCT4, belongs to the POU transcription factor family. It is well documented that POU5F1 is highly expressed in many cancer types, and nucleus POU5F1 contributes to tumorigenicity and maintaining stemness of cancer stem cells. *C-myc* is a well-known proto-oncogene, which causally contributed to the genesis of up to 30% of all human cancers [42]. Nucleus localization of c-myc plays an important role in the oncogenic functions of c-myc. In addition, KPNA2 has been reported to mediate the nucleus transport of both POU5F1 and c-myc. We found that both overexpression of miR-139 and knockdown of KPNA2 led to decreased nucleus localization of POU5F1 and c-myc and that KPNA2 directly binds to POU5F1 and c-myc. Therefore, these results suggested that miR-139/KPNA2 axis might regulate HCC growth potentially via regulating the nucleus transportation of important cancer-related factors, such as POU5F1 and c-myc.

It is well known that HCC can be caused by chemicals from environments and viruses such as HBV and HCV, and the genetic alterations between these two types of HCC vary widely. When we collected the clinical tissues and use the LICH database, however, we did not separate the HCC cases based on their causes, because sometimes it is hard to distinguish the cause of the patients as chemical or virus, some patients may even have both causes. In our future studies, we will try to collect samples from patients caused by chemicals and virus separately for experiments. In addition, the correlation between miR-139 or KPNA2 expression and HBV or HCV remain largely unknown. Mourad et al. has reported that circulating miR-139 level was down-regulated in HCV-induced HCC, but they did not investigate the role of miR-139 in this type of HCC [43]. In the future study, we will investigate the roles of miR-139 and KPNA2 in HCC formation and development under the background of HBV or HCV infection.

## Conclusion

In sum, we verified that miR-139 plays a tumor suppressive role in HCC and low expression of miR-139 is associated with poor prognosis. KPNA2 is a direct target of miR-139 and it can promote HCC growth likely via regulating nucleus transport of cancer-related proteins. And the tumor suppressive effect of miR-139 is partially through down-regulating the expression KPNA2. This study revealed the importance of the miR-139/KPNA2 axis in formation and development of HCC. It extended our understanding in the molecular mechanism for HCC development and suggested that miR-139/KPNA2 axis represents a candidate therapeutic target for HCC treatment.

## Abbreviations

3′ UTR: 3′untranslated region
5-AZA: 5-Azacytidine
CRC: Colorectal cancer
EMT: Epithelial-to-mesenchymal transition
HCC: Hepatocellular carcinoma
miRNA: MicroRNA
MTT: 3-(4,5-dimethylthiazol-2-yl)-2,5-diphenyltetrazolium bromide
NLS: Nuclear localization signal
OS: Overall survival
TCGA: The Cancer Genome Atlas
TSA: Trichostatin A
